# 4-[(*E*)-4-Bromo­benzyl­ideneamino]-3-[1-(4-isobutyl­phen­yl)eth­yl]-1*H*-1,2,4-triazole-5(4*H*)-thione

**DOI:** 10.1107/S1600536809018030

**Published:** 2009-05-20

**Authors:** Hoong-Kun Fun, Samuel Robinson Jebas, K. V. Sujith, Balakrishna Kalluraya

**Affiliations:** a X-ray Crystallography Unit, School of Physics, Universiti Sains Malaysia, 11800 USM, Penang, Malaysia; b Department of studies in Chemistry, Mangalore University, Mangalagangotri, Mangalore 574 199, India

## Abstract

In the title compound, C_21_H_23_BrN_4_S, the 4-bromo­benzyl­idene group is disordered over two orientations with occupancies of 0.504 (5) and 0.496 (5). One of the methyl groups of the isobutyl unit is disordered over two sites with occupancies of 0.751 (19) and 0.249 (19). The benzene rings of the isobutylphenyl and bromo­phenyl (major disorder component) groups form dihedral angles of 71.63 (11) and 21.8 (3)°, respectively, with the triazole ring. In the crystal, centrosymmetrically related mol­ecules exist as centrosymmetric N—H⋯S hydrogen-bonded dimers.

## Related literature

For the pharmaceutical applications of triazole derivatives, see: Al-Soud *et al.* (2003 ▶); Almasirad *et al.* (2004 ▶); Amir & Shikha (2004 ▶); Demirbas *et al.* (2004 ▶); Holla *et al.* (2003 ▶); Kawashima *et al.* (1987 ▶); Zitouni *et al.* (2005 ▶); Walczak *et al.* (2004 ▶); For bond-length data, see: Allen *et al.* (1987 ▶). For related structures, see: Fun *et al.* (2008*a* ▶,*b* ▶). For the stability of the temperature controller used in the data collection, see: Cosier & Glazer (1986 ▶).
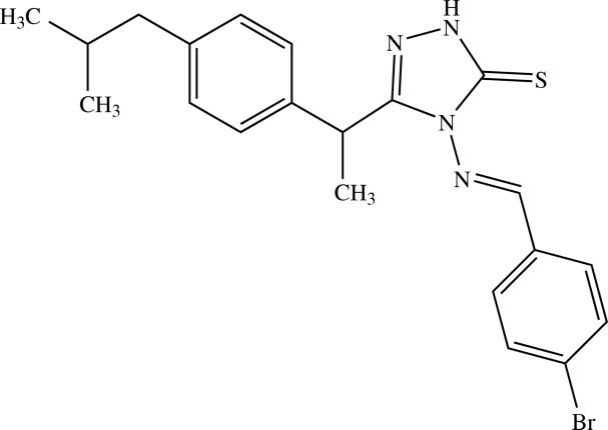


## Experimental

### 

#### Crystal data

C_21_H_23_BrN_4_S
*M* *_r_* = 443.40
Triclinic, 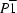

*a* = 5.5791 (2) Å
*b* = 11.3052 (3) Å
*c* = 17.3688 (4) Å
α = 75.421 (1)°
β = 86.614 (1)°
γ = 79.616 (1)°
*V* = 1042.75 (5) Å^3^
*Z* = 2
Mo *K*α radiation
μ = 2.08 mm^−1^
*T* = 100 K
0.27 × 0.17 × 0.05 mm

#### Data collection

Bruker SMART APEXII CCD area-detector diffractometer
Absorption correction: multi-scan (*SADABS*; Bruker, 2005 ▶) *T*_min_ = 0.602, *T*_max_ = 0.908
26698 measured reflections
8529 independent reflections
5907 reflections with *I* > 2σ(*I*)
*R*_int_ = 0.029

#### Refinement

*R*[*F* ^2^ > 2σ(*F* ^2^)] = 0.052
*wR*(*F* ^2^) = 0.141
*S* = 1.05
8529 reflections
331 parameters
44 restraints
H atoms treated by a mixture of independent and constrained refinement
Δρ_max_ = 0.67 e Å^−3^
Δρ_min_ = −0.83 e Å^−3^

### 

Data collection: *APEX2* (Bruker, 2005 ▶); cell refinement: *SAINT* (Bruker, 2005 ▶); data reduction: *SAINT*; program(s) used to solve structure: *SHELXTL* (Sheldrick, 2008 ▶); program(s) used to refine structure: *SHELXTL*; molecular graphics: *SHELXTL*; software used to prepare material for publication: *SHELXTL* and *PLATON* (Spek, 2009 ▶).

## Supplementary Material

Crystal structure: contains datablocks global, I. DOI: 10.1107/S1600536809018030/ci2795sup1.cif

Structure factors: contains datablocks I. DOI: 10.1107/S1600536809018030/ci2795Isup2.hkl

Additional supplementary materials:  crystallographic information; 3D view; checkCIF report

## Figures and Tables

**Table 1:** Hydrogen-bond geometry (Å, °)

*D*—H⋯*A*	*D*—H	H⋯*A*	*D*⋯*A*	*D*—H⋯*A*
N1—H1*N*1⋯S1^i^	0.91 (4)	2.35 (4)	3.2582 (18)	175 (4)

Symmetry code: (i) 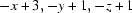.

**Fig. 1.**
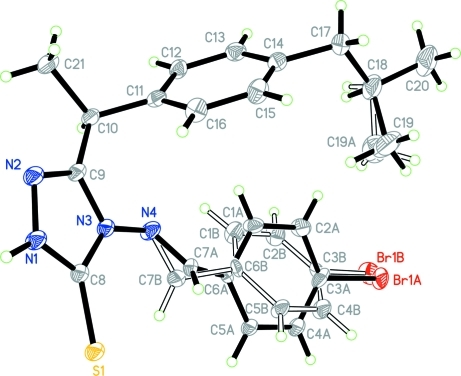
The molecular structure of the title compound, showing 50% probability displacement ellipsoids and the atom-numbering scheme. Both disorder components are shown.

**Fig. 2.**
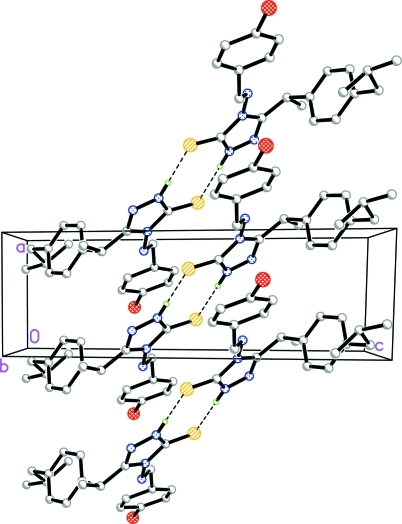
The crystal packing of the title compound, viewed along the b axis. Dashed lines indicate N—H···S hydrogen bonds. Only major disorder components are shown. For clarity, H atoms not involved in hydrogen bonding have been removed.

## supplementary crystallographic information

### Comment

Some degree of respectability has been bestowed on 1,2,4-triazole derivatives due to their antibacterial, antifungal (Zitouni *et al.*, 2005), antitubercular (Walczak *et al.*, 2004), anticancer (Holla *et al.*, 2003), antitumor (Al-Soud *et al.*, 2003), anticonvulsant (Almasirad *et al.*, 2004), anti-inflammatory, and analgesic properties (Amir & Shikha, 2004). Certain 1,2,4-triazoles also find applications in the preparation of photographic plates, polymers, and as analytical agents (Kawashima *et al.*, 1987). Similarly Schiff base derivatives of 1,2,4-triazol-5-ones have been found to possess antitumor activity (Demirbas *et al.*, 2004). In our earlier studies, we have reported the crystal structure of heterocyclic compounds containing both the ibuprofen and 1,2,4- triazole fragments (Fun *et al.*, 2008*a*,b). Prompted by these observations and in continuation of our interest in the synthesis of chemically and biologically important heterocycles, we synthesized the title compound and report here its crystal structure.

Bond lengths (Allen *et al.*, 1987) and angles are normal. The (4-bromophenyl)methylidene group is disordered over two orientations. The C11-C16 benzene ring forms a dihedral angle of 71.63 (11)° with the triazole ring (N1-N3/C8/C9). The dihedral angle between the C1A-C6A and N1-N3/C8/C9 rings is 21.8 (3)°.

The crystal packing (Fig 2) is consolidated by intermolecular N—H···S hygrogen bonds. These hydrogen bonds link centrosymmetrically related molecules into dimers.

### Experimental

The title Schiff base compound was obtained by refluxing 4-amino-5-[1-(4-isobutylphenyl)ethyl]-4*H*-1,2,4-triazole-3-thiol (0.01 mol) and 4-bromobenzaldehyde (0.01 mol) in ethanol (50 ml) with 3 drops of concentrated sulfuric acid for 3 h. The solid product obtained was collected by filtration, washed with ethanol and dried. It was then recrystallized using ethanol. Single crystals suitable for X-ray analysis were obtained by slow evaporation of an ethanol solution.

### Refinement

The (4-bromophenyl)methylidene group is disordered over two orientations with occupancies of 0.504 (5) and 0.496 (5), whereas, one of the methyl groups of the isobutyl unit is disordered over two sites with occupancies of 0.751 (19) and 0.249 (19). The corresponding bond distances in major and minor disorder components were restrained to be equal. The displacement parameters of atoms C19 and C19A were restrained to approximate isotropic behaviour. The N bound H atom was located in a difference map and was refined freely. C-bound H atoms were positioned geometrically [C–H = 0.93–0.98 Å] and refined using a riding model, with *U*_iso_(H) = 1.2*U*_eq_(C) and 1.5*U*_eq_(methyl C). A rotating–group model was used for the methyl groups.

### Crystal data

**Table:**

C_21_H_23_BrN_4_S	*Z* = 2
*M**_r_* = 443.40	*F*(000) = 456
Triclinic, *P*1	*D*_x_ = 1.412 Mg m^−^^3^
Hall symbol: -P 1	Mo *K*α radiation, λ = 0.71073 Å
*a* = 5.5791 (2) Å	Cell parameters from 9940 reflections
*b* = 11.3052 (3) Å	θ = 2.7–33.0°
*c* = 17.3688 (4) Å	µ = 2.08 mm^−^^1^
α = 75.421 (1)°	*T* = 100 K
β = 86.614 (1)°	Plate, colourless
γ = 79.616 (1)°	0.27 × 0.17 × 0.05 mm
*V* = 1042.75 (5) Å^3^	

### Data collection

**Table:**

Bruker SMART APEXII CCD area-detector diffractometer	8529 independent reflections
Radiation source: fine-focus sealed tube	5907 reflections with *I* > 2σ(*I*)
graphite	*R*_int_ = 0.029
φ and ω scans	θ_max_ = 34.2°, θ_min_ = 2.4°
Absorption correction: multi-scan (*SADABS*; Bruker, 2005)	*h* = −8→8
*T*_min_ = 0.602, *T*_max_ = 0.908	*k* = −17→17
26698 measured reflections	*l* = −27→27

### Refinement

**Table:**

Refinement on *F*^2^	Primary atom site location: structure-invariant direct methods
Least-squares matrix: full	Secondary atom site location: difference Fourier map
*R*[*F*^2^ > 2σ(*F*^2^)] = 0.052	Hydrogen site location: inferred from neighbouring sites
*wR*(*F*^2^) = 0.141	H atoms treated by a mixture of independent and constrained refinement
*S* = 1.05	*w* = 1/[σ^2^(*F*_o_^2^) + (0.0732*P*)^2^ + 0.4173*P*] where *P* = (*F*_o_^2^ + 2*F*_c_^2^)/3
8529 reflections	(Δ/σ)_max_ = 0.001
331 parameters	Δρ_max_ = 0.67 e Å^−^^3^
44 restraints	Δρ_min_ = −0.83 e Å^−^^3^

### Special details

**Table:**

Experimental. The crystal was placed in the cold stream of an Oxford Cyrosystems Cobra open-flow nitrogen cryostat (Cosier & Glazer, 1986) operating at 100.0 (1) K.
Geometry. All e.s.d.'s (except the e.s.d. in the dihedral angle between two l.s. planes) are estimated using the full covariance matrix. The cell e.s.d.'s are taken into account individually in the estimation of e.s.d.'s in distances, angles and torsion angles; correlations between e.s.d.'s in cell parameters are only used when they are defined by crystal symmetry. An approximate (isotropic) treatment of cell e.s.d.'s is used for estimating e.s.d.'s involving l.s. planes.
Refinement. Refinement of *F*^2^ against ALL reflections. The weighted *R*-factor *wR* and goodness of fit *S* are based on *F*^2^, conventional *R*-factors *R* are based on *F*, with *F* set to zero for negative *F*^2^. The threshold expression of *F*^2^ > σ(*F*^2^) is used only for calculating *R*-factors(gt) *etc*. and is not relevant to the choice of reflections for refinement. *R*-factors based on *F*^2^ are statistically about twice as large as those based on *F*, and *R*- factors based on ALL data will be even larger.

### Fractional atomic coordinates and isotropic or equivalent isotropic displacement parameters (Å^2^)

**Table:**

	*x*	*y*	*z*	*U*_iso_*/*U*_eq_	Occ. (<1)
S1	1.28609 (9)	0.34264 (4)	0.51104 (3)	0.01997 (11)	
N1	1.3036 (3)	0.56195 (15)	0.40486 (11)	0.0218 (3)	
N2	1.1990 (3)	0.64191 (15)	0.33670 (10)	0.0210 (3)	
N3	1.0344 (3)	0.47201 (14)	0.37298 (10)	0.0179 (3)	
N4	0.8770 (3)	0.39601 (15)	0.36230 (10)	0.0207 (3)	
Br1A	0.3846 (3)	−0.10655 (16)	0.32563 (14)	0.0250 (2)	0.504 (5)
C1A	0.6489 (11)	0.2199 (4)	0.3123 (3)	0.0230 (10)	0.504 (5)
H1A	0.6584	0.2931	0.2740	0.028*	0.504 (5)
C2A	0.5425 (10)	0.1269 (4)	0.2937 (3)	0.0257 (10)	0.504 (5)
H2A	0.4850	0.1376	0.2428	0.031*	0.504 (5)
C3A	0.524 (3)	0.0185 (11)	0.3521 (7)	0.023 (2)	0.504 (5)
C4A	0.6099 (8)	0.0001 (3)	0.4279 (2)	0.0222 (9)	0.504 (5)
H4A	0.5976	−0.0728	0.4661	0.027*	0.504 (5)
C5A	0.7157 (8)	0.0922 (3)	0.4464 (2)	0.0200 (8)	0.504 (5)
H5A	0.7704	0.0804	0.4977	0.024*	0.504 (5)
C6A	0.7418 (14)	0.2021 (7)	0.3895 (4)	0.0140 (13)	0.504 (5)
C7A	0.9410 (9)	0.2829 (4)	0.3857 (3)	0.0164 (8)	0.504 (5)
H7A	1.1007	0.2478	0.4003	0.020*	0.504 (5)
Br1B	0.3266 (4)	−0.0828 (2)	0.32652 (14)	0.0315 (3)	0.496 (5)
C1B	0.5581 (10)	0.2422 (5)	0.3354 (4)	0.0251 (10)	0.496 (5)
H1B	0.4996	0.3270	0.3207	0.030*	0.496 (5)
C2B	0.4229 (9)	0.1591 (4)	0.3211 (3)	0.0259 (10)	0.496 (5)
H2B	0.2742	0.1874	0.2955	0.031*	0.496 (5)
C3B	0.511 (2)	0.0331 (10)	0.3453 (7)	0.019 (2)	0.496 (5)
C4B	0.7310 (8)	−0.0122 (4)	0.3842 (3)	0.0227 (9)	0.496 (5)
H4B	0.7865	−0.0972	0.4007	0.027*	0.496 (5)
C5B	0.8665 (7)	0.0715 (3)	0.3982 (2)	0.0205 (9)	0.496 (5)
H5B	1.0132	0.0433	0.4250	0.025*	0.496 (5)
C6B	0.7815 (16)	0.1976 (8)	0.3717 (4)	0.0177 (15)	0.496 (5)
C7B	0.8642 (10)	0.2913 (4)	0.4107 (3)	0.0195 (9)	0.496 (5)
H7B	0.9015	0.2734	0.4643	0.023*	0.496 (5)
C8	1.2086 (4)	0.45804 (17)	0.42984 (11)	0.0183 (3)	
C9	1.0332 (4)	0.58544 (17)	0.31907 (12)	0.0186 (3)	
C10	0.8618 (4)	0.63522 (17)	0.25106 (11)	0.0182 (3)	
H10	0.6956	0.6424	0.2732	0.022*	
C11	0.8818 (3)	0.55032 (17)	0.19481 (11)	0.0182 (3)	
C12	0.6815 (4)	0.55549 (18)	0.14931 (12)	0.0202 (4)	
H12	0.5389	0.6103	0.1541	0.024*	
C13	0.6911 (4)	0.48005 (19)	0.09682 (12)	0.0231 (4)	
H13	0.5545	0.4853	0.0671	0.028*	
C14	0.9017 (4)	0.39637 (18)	0.08781 (12)	0.0216 (4)	
C15	1.1011 (4)	0.3927 (2)	0.13315 (14)	0.0248 (4)	
H15	1.2441	0.3382	0.1283	0.030*	
C16	1.0928 (4)	0.46833 (19)	0.18572 (13)	0.0236 (4)	
H16	1.2299	0.4639	0.2150	0.028*	
C17	0.9038 (5)	0.3124 (2)	0.03308 (14)	0.0269 (4)	
H17A	1.0714	0.2834	0.0194	0.032*	
H17B	0.8221	0.3598	−0.0157	0.032*	
C18	0.7800 (5)	0.1999 (2)	0.06865 (16)	0.0336 (5)	
H18A	0.6260	0.2265	0.0946	0.040*	0.751 (19)
H18B	0.6190	0.2385	0.0806	0.040*	0.249 (19)
C19	0.9578 (16)	0.1074 (4)	0.1326 (3)	0.0482 (17)	0.751 (19)
H19A	0.8860	0.0355	0.1564	0.072*	0.751 (19)
H19B	0.9859	0.1477	0.1729	0.072*	0.751 (19)
H19C	1.1099	0.0828	0.1074	0.072*	0.751 (19)
C19A	0.833 (5)	0.1155 (12)	0.1451 (7)	0.045 (4)	0.249 (19)
H19D	0.6845	0.1088	0.1756	0.068*	0.249 (19)
H19E	0.9431	0.1459	0.1728	0.068*	0.249 (19)
H19F	0.9065	0.0353	0.1380	0.068*	0.249 (19)
C20	0.7304 (6)	0.1367 (3)	0.0047 (2)	0.0443 (7)	
H20A	0.6351	0.1960	−0.0366	0.066*	
H20B	0.6427	0.0703	0.0278	0.066*	
H20C	0.8823	0.1040	−0.0174	0.066*	
C21	0.9022 (5)	0.76614 (19)	0.20644 (14)	0.0282 (4)	
H21A	0.8821	0.8187	0.2428	0.042*	
H21B	0.7857	0.7995	0.1646	0.042*	
H21C	1.0640	0.7620	0.1840	0.042*	
H1N1	1.424 (7)	0.584 (4)	0.429 (2)	0.058 (10)*	

### Atomic displacement parameters (Å^2^)

**Table:**

	*U*^11^	*U*^22^	*U*^33^	*U*^12^	*U*^13^	*U*^23^
S1	0.0262 (2)	0.01396 (19)	0.0200 (2)	−0.00712 (16)	−0.00585 (17)	−0.00092 (16)
N1	0.0275 (8)	0.0159 (7)	0.0224 (8)	−0.0096 (6)	−0.0078 (7)	0.0004 (6)
N2	0.0268 (8)	0.0164 (7)	0.0200 (8)	−0.0085 (6)	−0.0040 (6)	−0.0006 (6)
N3	0.0230 (7)	0.0137 (7)	0.0179 (7)	−0.0067 (6)	−0.0035 (6)	−0.0022 (5)
N4	0.0228 (8)	0.0146 (7)	0.0244 (8)	−0.0080 (6)	−0.0057 (6)	0.0001 (6)
Br1A	0.0305 (6)	0.0176 (4)	0.0322 (3)	−0.0109 (3)	−0.0032 (4)	−0.0103 (3)
C1A	0.032 (3)	0.019 (2)	0.018 (2)	−0.0129 (19)	−0.0069 (18)	0.0021 (15)
C2A	0.035 (3)	0.0224 (19)	0.023 (2)	−0.0130 (18)	−0.0103 (19)	−0.0035 (16)
C3A	0.026 (4)	0.011 (2)	0.030 (4)	−0.004 (2)	0.005 (3)	−0.001 (2)
C4A	0.033 (2)	0.0124 (15)	0.0231 (19)	−0.0097 (14)	−0.0019 (17)	−0.0024 (13)
C5A	0.029 (2)	0.0126 (15)	0.0186 (17)	−0.0080 (13)	−0.0031 (14)	−0.0011 (13)
C6A	0.023 (3)	0.0115 (19)	0.009 (3)	−0.0039 (17)	−0.003 (2)	−0.0036 (19)
C7A	0.021 (2)	0.0120 (16)	0.017 (2)	−0.0037 (15)	−0.0023 (16)	−0.0046 (15)
Br1B	0.0392 (8)	0.0315 (8)	0.0318 (3)	−0.0229 (5)	−0.0061 (6)	−0.0093 (5)
C1B	0.022 (2)	0.021 (2)	0.033 (3)	−0.0042 (18)	−0.008 (2)	−0.0075 (19)
C2B	0.022 (2)	0.027 (2)	0.033 (2)	−0.0072 (17)	−0.0047 (18)	−0.0108 (18)
C3B	0.026 (4)	0.022 (4)	0.017 (3)	−0.014 (3)	−0.006 (2)	−0.010 (3)
C4B	0.027 (2)	0.0158 (17)	0.027 (2)	−0.0086 (14)	−0.0062 (17)	−0.0043 (15)
C5B	0.0243 (18)	0.0125 (15)	0.025 (2)	−0.0066 (13)	−0.0076 (15)	−0.0015 (13)
C6B	0.029 (3)	0.015 (2)	0.012 (3)	−0.0049 (19)	0.003 (2)	−0.009 (2)
C7B	0.025 (2)	0.0170 (19)	0.017 (2)	−0.0063 (17)	−0.0018 (17)	−0.0023 (16)
C8	0.0234 (9)	0.0144 (7)	0.0178 (8)	−0.0060 (6)	−0.0032 (7)	−0.0027 (6)
C9	0.0238 (9)	0.0130 (7)	0.0187 (8)	−0.0062 (6)	−0.0014 (7)	−0.0011 (6)
C10	0.0219 (8)	0.0130 (7)	0.0188 (8)	−0.0040 (6)	−0.0035 (7)	−0.0006 (6)
C11	0.0196 (8)	0.0144 (7)	0.0191 (8)	−0.0051 (6)	−0.0027 (6)	0.0004 (6)
C12	0.0208 (8)	0.0159 (8)	0.0228 (9)	0.0005 (6)	−0.0046 (7)	−0.0044 (7)
C13	0.0260 (9)	0.0210 (9)	0.0215 (9)	−0.0051 (7)	−0.0066 (7)	−0.0020 (7)
C14	0.0273 (9)	0.0173 (8)	0.0206 (9)	−0.0076 (7)	0.0028 (7)	−0.0032 (7)
C15	0.0192 (9)	0.0219 (9)	0.0329 (11)	−0.0015 (7)	0.0022 (8)	−0.0081 (8)
C16	0.0182 (8)	0.0233 (9)	0.0299 (10)	−0.0040 (7)	−0.0021 (7)	−0.0071 (8)
C17	0.0367 (11)	0.0202 (9)	0.0252 (10)	−0.0054 (8)	0.0016 (9)	−0.0082 (8)
C18	0.0443 (13)	0.0196 (10)	0.0392 (13)	−0.0108 (9)	0.0066 (11)	−0.0091 (9)
C19	0.064 (4)	0.0223 (16)	0.052 (2)	−0.0045 (18)	−0.012 (2)	0.0026 (15)
C19A	0.058 (6)	0.035 (5)	0.045 (5)	−0.015 (4)	0.010 (4)	−0.011 (4)
C20	0.0526 (17)	0.0268 (12)	0.0591 (18)	−0.0079 (11)	−0.0076 (14)	−0.0186 (12)
C21	0.0406 (12)	0.0167 (9)	0.0258 (10)	−0.0076 (8)	−0.0073 (9)	0.0011 (7)

### Geometric parameters (Å, °)

**Table:**

S1—C8	1.6794 (19)	C9—C10	1.494 (3)
N1—C8	1.338 (2)	C10—C11	1.520 (3)
N1—N2	1.383 (2)	C10—C21	1.538 (3)
N1—H1N1	0.91 (4)	C10—H10	0.98
N2—C9	1.305 (2)	C11—C16	1.389 (3)
N3—C9	1.384 (2)	C11—C12	1.391 (3)
N3—N4	1.384 (2)	C12—C13	1.389 (3)
N3—C8	1.387 (2)	C12—H12	0.93
N4—C7A	1.232 (5)	C13—C14	1.398 (3)
N4—C7B	1.279 (5)	C13—H13	0.93
Br1A—C3A	1.891 (13)	C14—C15	1.389 (3)
C1A—C2A	1.408 (5)	C14—C17	1.501 (3)
C1A—C6A	1.419 (7)	C15—C16	1.393 (3)
C1A—H1A	0.93	C15—H15	0.93
C2A—C3A	1.397 (11)	C16—H16	0.93
C2A—H2A	0.93	C17—C18	1.533 (3)
C3A—C4A	1.381 (11)	C17—H17A	0.97
C4A—C5A	1.395 (5)	C17—H17B	0.97
C4A—H4A	0.93	C18—C19A	1.438 (12)
C5A—C6A	1.405 (8)	C18—C20	1.526 (4)
C5A—H5A	0.93	C18—C19	1.576 (5)
C6A—C7A	1.549 (8)	C18—H18A	0.98
C7A—H7A	0.93	C18—H18B	0.96
Br1B—C3B	1.903 (10)	C19—H19A	0.96
C1B—C2B	1.381 (6)	C19—H19B	0.96
C1B—C6B	1.383 (9)	C19—H19C	0.96
C1B—H1B	0.93	C19A—H19D	0.96
C2B—C3B	1.385 (10)	C19A—H19E	0.96
C2B—H2B	0.93	C19A—H19F	0.96
C3B—C4B	1.386 (10)	C20—H20A	0.96
C4B—C5B	1.386 (5)	C20—H20B	0.96
C4B—H4B	0.93	C20—H20C	0.96
C5B—C6B	1.386 (9)	C21—H21A	0.96
C5B—H5B	0.93	C21—H21B	0.96
C6B—C7B	1.538 (9)	C21—H21C	0.96
C7B—H7B	0.93		
			
C8—N1—N2	114.19 (16)	C21—C10—H10	107.6
C8—N1—H1N1	126 (2)	C16—C11—C12	118.05 (18)
N2—N1—H1N1	120 (2)	C16—C11—C10	123.04 (17)
C9—N2—N1	104.04 (15)	C12—C11—C10	118.90 (17)
C9—N3—N4	119.15 (15)	C13—C12—C11	121.08 (19)
C9—N3—C8	108.45 (15)	C13—C12—H12	119.5
N4—N3—C8	132.40 (15)	C11—C12—H12	119.5
C7A—N4—N3	118.1 (3)	C12—C13—C14	121.30 (18)
C7B—N4—N3	122.6 (3)	C12—C13—H13	119.3
C2A—C1A—C6A	120.1 (5)	C14—C13—H13	119.3
C2A—C1A—H1A	119.9	C15—C14—C13	117.07 (19)
C6A—C1A—H1A	119.9	C15—C14—C17	122.7 (2)
C3A—C2A—C1A	119.6 (6)	C13—C14—C17	120.17 (19)
C3A—C2A—H2A	120.2	C14—C15—C16	121.89 (19)
C1A—C2A—H2A	120.2	C14—C15—H15	119.1
C4A—C3A—C2A	121.2 (9)	C16—C15—H15	119.1
C4A—C3A—Br1A	119.7 (7)	C11—C16—C15	120.60 (19)
C2A—C3A—Br1A	119.1 (7)	C11—C16—H16	119.7
C3A—C4A—C5A	119.2 (6)	C15—C16—H16	119.7
C3A—C4A—H4A	120.4	C14—C17—C18	113.68 (19)
C5A—C4A—H4A	120.4	C14—C17—H17A	108.8
C4A—C5A—C6A	121.8 (4)	C18—C17—H17A	108.8
C4A—C5A—H5A	119.1	C14—C17—H17B	108.8
C6A—C5A—H5A	119.1	C18—C17—H17B	108.8
C5A—C6A—C1A	118.1 (6)	H17A—C17—H17B	107.7
C5A—C6A—C7A	127.4 (6)	C19A—C18—C20	113.6 (6)
C1A—C6A—C7A	109.9 (5)	C19A—C18—C17	125.0 (8)
N4—C7A—C6A	116.6 (5)	C20—C18—C17	111.2 (2)
N4—C7A—H7A	121.7	C20—C18—C19	110.1 (3)
C6A—C7A—H7A	121.7	C17—C18—C19	107.0 (3)
C2B—C1B—C6B	119.2 (5)	C19A—C18—H18A	83.7
C2B—C1B—H1B	120.4	C20—C18—H18A	109.5
C6B—C1B—H1B	120.4	C17—C18—H18A	109.5
C1B—C2B—C3B	119.3 (5)	C19—C18—H18A	109.5
C1B—C2B—H2B	120.3	C19A—C18—H18B	98.3
C3B—C2B—H2B	120.3	C20—C18—H18B	101.9
C2B—C3B—C4B	121.8 (7)	C17—C18—H18B	102.0
C2B—C3B—Br1B	119.9 (6)	C19—C18—H18B	124.2
C4B—C3B—Br1B	118.3 (7)	C18—C19—H19A	109.5
C5B—C4B—C3B	118.7 (5)	C18—C19—H19B	109.5
C5B—C4B—H4B	120.6	H19A—C19—H19B	109.5
C3B—C4B—H4B	120.6	C18—C19—H19C	109.5
C6B—C5B—C4B	119.4 (5)	H19A—C19—H19C	109.5
C6B—C5B—H5B	120.3	H19B—C19—H19C	109.5
C4B—C5B—H5B	120.3	C18—C19A—H19D	109.5
C1B—C6B—C5B	121.5 (7)	C18—C19A—H19E	109.5
C1B—C6B—C7B	112.5 (6)	H19D—C19A—H19E	109.5
C5B—C6B—C7B	121.0 (6)	C18—C19A—H19F	109.5
N4—C7B—C6B	113.6 (5)	H19D—C19A—H19F	109.5
N4—C7B—H7B	123.2	H19E—C19A—H19F	109.5
C6B—C7B—H7B	123.2	C18—C20—H20A	109.5
N1—C8—N3	102.67 (15)	C18—C20—H20B	109.5
N1—C8—S1	127.12 (15)	H20A—C20—H20B	109.5
N3—C8—S1	130.21 (14)	C18—C20—H20C	109.5
N2—C9—N3	110.62 (17)	H20A—C20—H20C	109.5
N2—C9—C10	125.50 (16)	H20B—C20—H20C	109.5
N3—C9—C10	123.88 (16)	C10—C21—H21A	109.5
C9—C10—C11	112.76 (16)	C10—C21—H21B	109.5
C9—C10—C21	109.82 (16)	H21A—C21—H21B	109.5
C11—C10—C21	111.13 (17)	C10—C21—H21C	109.5
C9—C10—H10	107.6	H21A—C21—H21C	109.5
C11—C10—H10	107.6	H21B—C21—H21C	109.5
			
C8—N1—N2—C9	0.1 (2)	N2—N1—C8—S1	−178.50 (16)
C9—N3—N4—C7A	149.7 (3)	C9—N3—C8—N1	−1.7 (2)
C8—N3—N4—C7A	−29.4 (4)	N4—N3—C8—N1	177.6 (2)
C9—N3—N4—C7B	−178.4 (4)	C9—N3—C8—S1	177.80 (17)
C8—N3—N4—C7B	2.4 (5)	N4—N3—C8—S1	−2.9 (3)
C6A—C1A—C2A—C3A	1.5 (12)	N1—N2—C9—N3	−1.2 (2)
C1A—C2A—C3A—C4A	−0.6 (17)	N1—N2—C9—C10	178.08 (19)
C1A—C2A—C3A—Br1A	−179.1 (7)	N4—N3—C9—N2	−177.49 (17)
C2A—C3A—C4A—C5A	0.6 (16)	C8—N3—C9—N2	1.9 (2)
Br1A—C3A—C4A—C5A	179.0 (6)	N4—N3—C9—C10	3.2 (3)
C3A—C4A—C5A—C6A	−1.4 (11)	C8—N3—C9—C10	−177.41 (19)
C4A—C5A—C6A—C1A	2.2 (9)	N2—C9—C10—C11	122.2 (2)
C4A—C5A—C6A—C7A	−150.8 (6)	N3—C9—C10—C11	−58.6 (3)
C2A—C1A—C6A—C5A	−2.3 (10)	N2—C9—C10—C21	−2.3 (3)
C2A—C1A—C6A—C7A	155.2 (6)	N3—C9—C10—C21	176.8 (2)
C7B—N4—C7A—C6A	60.1 (8)	C9—C10—C11—C16	−26.2 (3)
N3—N4—C7A—C6A	167.3 (4)	C21—C10—C11—C16	97.6 (2)
C5A—C6A—C7A—N4	−143.9 (6)	C9—C10—C11—C12	154.71 (18)
C1A—C6A—C7A—N4	61.2 (8)	C21—C10—C11—C12	−81.5 (2)
C6B—C1B—C2B—C3B	1.6 (11)	C16—C11—C12—C13	0.6 (3)
C1B—C2B—C3B—C4B	0.6 (16)	C10—C11—C12—C13	179.79 (18)
C1B—C2B—C3B—Br1B	179.7 (7)	C11—C12—C13—C14	0.0 (3)
C2B—C3B—C4B—C5B	−0.8 (15)	C12—C13—C14—C15	−0.4 (3)
Br1B—C3B—C4B—C5B	180.0 (6)	C12—C13—C14—C17	177.59 (19)
C3B—C4B—C5B—C6B	−1.1 (10)	C13—C14—C15—C16	0.3 (3)
C2B—C1B—C6B—C5B	−3.7 (10)	C17—C14—C15—C16	−177.7 (2)
C2B—C1B—C6B—C7B	−158.9 (6)	C12—C11—C16—C15	−0.8 (3)
C4B—C5B—C6B—C1B	3.4 (9)	C10—C11—C16—C15	−179.91 (19)
C4B—C5B—C6B—C7B	156.6 (6)	C14—C15—C16—C11	0.3 (3)
C7A—N4—C7B—C6B	−63.2 (8)	C15—C14—C17—C18	99.4 (3)
N3—N4—C7B—C6B	−152.4 (4)	C13—C14—C17—C18	−78.5 (3)
C1B—C6B—C7B—N4	−57.0 (8)	C14—C17—C18—C19A	−51.5 (11)
C5B—C6B—C7B—N4	147.5 (6)	C14—C17—C18—C20	165.7 (2)
N2—N1—C8—N3	1.0 (2)	C14—C17—C18—C19	−74.0 (4)

### Hydrogen-bond geometry (Å, °)

**Table:**

*D*—H···*A*	*D*—H	H···*A*	*D*···*A*	*D*—H···*A*
N1—H1N1···S1^i^	0.91 (4)	2.35 (4)	3.2582 (18)	175 (4)

Symmetry codes: (i) −*x*+3, −*y*+1, −*z*+1.
